# The quality of working life: gap between perception and idealization impact of gender and status

**DOI:** 10.3389/fpsyg.2023.1112737

**Published:** 2023-05-19

**Authors:** Edith Salès-Wuillemin, Brigitte Minondo-Kaghad, Julien Chappé, Morgan Gélin, Adrien Dolard

**Affiliations:** ^1^Université de Bourgogne, Dijon, France; ^2^Laboratoire Psy-DREPI, Psychologie Dynamiques Relationnelles Et Processus Identitaires, Dijon, France

**Keywords:** quality of working life, gender, management, QUALTRA-Scale, perception, expectations

## Abstract

**Background:**

Many studies show the existence of gender inequalities at work. For example, in France, only 37% of women have a managerial role, which is far from parity. Among these gender inequalities, the present study considers the Quality of Working Life (QWL) for women and managers.

**Method:**

This study measures the Quality of Working Life (QWL) perceived by individuals according to their gender (Women vs. Men) and their status (Managers vs. Co-workers). A questionnaire was distributed to 1,321 employees. It comprised two scales: the WRQoL scale and the QUALTRA-Scale. The QUALTRA-Scale permits the calculation of an index *δ* that measures the gap between the *ideal* QWL and the *perceived* QWL.

**Results:**

The ANOVA (2×2) revealed an impact of status on the perceived QWL on both scales. There was no gender effect. However, there was an effect of both gender and status on the index *δ* of the QUALTRA-Scale. In particular, for the Women group, *δ* was higher for the Social Relationships at Work whereas for the Co-workers, it was higher for the Work Environment.

**Conclusion:**

These results are discussed, highlighting the value of measuring the *ideal* QWL as a reference point for assessing the QWL.

## 1. Introduction

Work plays a major role in people’s lives and can have a negative impact on their personal lives if it is not a source of satisfaction. A recent report produced in France by the French Ministry of Equality between Women and Men (2022) shows gender inequalities in working conditions. Since the early 2000s, the analysis of working conditions has been included in the broader field of the study of the Quality of Working Life.

In June 2013, different professional organizations signed the French National Interprofessional Agreement (ANI, 2013) aimed at developing a policy of improved QWL and professional equality within the workplace. This agreement was followed by the decree of 15 April (ANI Decree, 2014), which made the provisions of the ANI mandatory. In March 2022, working conditions were placed directly at the heart of the QWL so it is now recommended to talk about the Quality of Life and Working Conditions.

There has been a great deal of research on the QWL (*cf.* Desrumaux et al., 2011; Althaus et al., 2016). It was initially developed as a subject of investigation in 1933 by Elton Mayo (e.g., Mayo, 1945) in the industrial environment. Later, Porter and Lawler (2013) emphasized that job satisfaction is a central factor in the QWL. More recent works show that a good QWL is a source of commitment and productivity (e.g., Horst et al., 2014).

The QWL can be defined as a synthesis of working conditions experienced, which stimulates an employee’s job satisfaction. In other words, the concept of the QWL refers to various interactive factors that affect individual satisfaction at work (Warr et al., 1979; Sirgy et al., 2001). As underlined by Easton et al. (2013), it is important to define the core facets of working conditions, and more exactly the working environment experience, to understand the QWL satisfaction. Furthermore, these facets cannot be adequately explained in isolation; they need to be considered together (Van Laar et al., 2007). Different facets can then be taken into account, for example the home-work interface or support at work. It is also argued that causes (working conditions) and effects (QWL perception) must be distinguished in order to target an appropriate intervention in the workplace.

As a recent survey shows (IFOP, 2022), in France, only 37% of women have a managerial role, which is far from parity. Among gender inequalities, the present study considers the quality of working life for women and managers. As noted by Gospel (2003), are women less satisfied with their work, as well as having a lower status and a lower salary? Do women have particular expectations regarding their working conditions and, more generally, their career development? Studies that focus specifically on the QWL of women and on the impact of their status as managers are very rare.

Several studies exploring gender differences in job satisfaction show that although women have poorer working conditions, job rewards and job values, there is no difference in general job satisfaction between women and men (e.g., Bokemeier and Lacy, 1987). Recently, Andrade et al. (2019), using data from the 2015 International Social Survey, examined the impact of extrinsic/intrinsic rewards, work relationships and work-life balance rewards on job satisfaction. They found the same results: there were no differences between women’s and men’s job satisfaction. Thus, some authors use the expression “gender-job satisfaction paradox” to describe this phenomenon. Among them, Mihaljov et al. (2021) try to elucidate this incongruity. By examining the dimensions of satisfaction, the authors show that there are different sources of satisfaction. Women are satisfied with their job if they have manager support through open and constructive communication. In contrast, men express satisfaction if the work organization provides them with opportunities for evolution and autonomy, and if their needs for self-realization are met.

These results show that it is necessary to nuance job satisfaction measurement by taking into account different sources of satisfaction; in other words, different dimensions of working conditions. These dimensions must be included when measuring the QWL.

Marcacine et al. (2019) show for example that women relate a better QWL in terms of the social relationships at work, but a worse QWL with regard to the work environment perception. Schoepke et al. (2004) are particularly interested in work organization and the characteristics of tasks (role ambiguity, decision control, work constraints) as well as working conditions. They show that women do not have lower scores than men for work organization perception. However, regarding working conditions, women report greater fatigue and a high state of tension, correlated with role ambiguity and low decision latitude. Turnover is also higher in women than in men.

Other research works focus on work organization, in particular the balance between one’s professional and personal lives (Eby et al., 2005; Hammer et al., 2011). A recent study (Chung and Van Der Lippe, 2020) shows the connection between the work/life balance on one hand and work organization, especially flexible working hours, on the other hand. It highlights that for women, compared to men, flexible working hours are not always an asset because they lead to some variability that can adversely affect the work/life balance, unlike fixed hours. Other studies show that flexible working hours can also affect the mental health of women (Sharma and Kapur, 2022).

There are only a few studies on the perception that managers have of their QWL. In her research, Britton (1997) underlines that there are factors that mediate the relationship between gender and job perceptions, particularly for managers. Wrzesniewski et al. (1997) show that the higher employees rise in the hierarchy, the higher are their expectations for their QWL. Mosadeghrad et al. (2011) demonstrate that for hospital staff, the most relevant predictors of the QWL are working conditions, such as risk management, job security and perceived stress. The study of Hashemi-Dehaghi and Sheikhtaheri (2014) deals with healthcare managers, who report quite a good overall QWL. In comparison, Dargahi et al. (2007) show that nurses report a very low QWL. Lastly, the study carried out in the industrial sector by Siron et al. (2013) reveals that managers are quite satisfied with their QWL, especially their communication with their co-workers. They consider the support of their hierarchy to be moderate and they report quite low scores for their work relationships with colleagues and co-workers. They also claim to have a great deal of stress.

To summarize, research shows that women report a lower QWL than men, especially concerning the work environment and work organization (role ambiguity and decision latitude), but a better QWL concerning social relationships at work, so a similar result could be expected in this study (Hypothesis 1). In the same way, studies show that managers report a better QWL than co-workers, especially concerning social relationships with their co-workers, but they state a lack of support from their hierarchy. Therefore, this result could be expected too (Hypothesis 2). However, in the workplace, women often have less qualified jobs, which could bias the results. In addition, the research carried out to date presents significant methodological limitations, due to the sample size being too small or not providing a specific comparison of Men/Women or Managers/Co-workers. In this case, the statistical analyses are carried out using a comparison point situated in the middle of the scale (Casper et al., 2007). It would thus be useful to confirm the results with a larger sample enabling these comparisons to be made.

Another question arises related to the scales used for measuring the different facets of the QWL. Various QWL scales are available (*cf.* Martel and Dupuis, 2006; Tavani et al., 2014). Among them, the Work-Related Quality of Life Scale, WRQoL, developed by Van Laar et al. (2007) and validated in French by Zenasni (2011), is widely used (e.g., Edwards et al., 2009; Easton et al., 2013; Sinval et al., 2019). The WRQoL scale comprises a range of six facets: (1) Job and Career Satisfaction; (2) General Well Being; (3) Home-Work Interface; (4) Stress at Work; (5) Control at Work; and (6) Working Conditions. The fundamental theoretical justification of this scale is based on the notion that these facets of work experience cannot be adequately explained in isolation. So, a closer inspection of the potential complex relationships between the six facets can help to understand how these factors influence one another. Due to its large number of uses and its dimensional structure, this scale was included in our study.

However, it does not comprise all the facets of the QWL mentioned in other studies comparing women/men or managers/co-workers QWL. For example, the perception of Work Organization, Social Relationships, or Support of the Hierarchy. That is why another scale was used in this study. The QUALTRA-Scale was developed by Salès-Wuillemin et al. (2016, 2017). This scale considers that QWL perception is related to the satisfaction that an individual experiences in the different dimensions of his/her working life. The QUALTRA-Scale comprises a range of six facets (or dimensions of working life): (1) Tasks and Work Organization; (2) Social Relationships at Work; (3) Professional Life-Personal Life balance; (4) Work Environment; (5) Professional Development; and (6) Recognition and Organizational Support. It is based on the idea that when individuals evaluate their satisfaction concerning their working environment, they do so from an ideal of life that they set for themselves. This ideal of life depends on their value system as well as their physical, social or cultural environment (*cf.* Gilgeous, 1998). In other words, the individual does not take, as a reference point, an absolute point represented by the highest number on the scale but positions him/herself in another universe that corresponds to an ideal to be achieved. In line with the analysis proposed by Gilgeous (1998), the authors assume that this reference point varies according to the individual and if it provides a goal to be reached, then by a feedback loop, it also makes it possible to assess one’s current quality of working life. This explains why it is useful to include these data in the measurement.

The use of a single Likert-type scale does not allow this reference point to be taken into account. It is therefore necessary to make two measurements: the first to assess the Perceived Quality of Working Life, as allowed by the existing scales; and the second to measure the Ideal Quality of Working Life and, more precisely, the importance of the dimension and the item considered to achieve this ideal, which current measurements cannot do. This is why in this study, a questionnaire was constructed including a scale that could measure the perceived QWL, the ideal QWL, as well as the difference between these two measurements. Thus, in the QUALTRA-Scale, each item has a double assessment measure: the current QWL (QWL-Perceived) and the expected QWL (QWL-Ideal).

## 2. Materials and methods

It was decided to question participants who work in very different organizations in order to highlight the invariants, independently of each person’s specific situation. To constitute a large sample, participants were contacted by 10 investigators *via* two social networks (Facebook, LinkedIn). The participants were questioned on an online platform and *via* a link sent through these two social and professional networks. An informed consent form, presenting the purpose of the study and an agreement to participate, was included at the beginning of the questionnaire. Before completing the questionnaire, respondents were asked to read the conditions and agree to participate in the study.

1,321 participants were questioned: 738 women and 307 men. 276 did not answer this question. The average age of respondents was 33.9 years (S.D. = 11.4; Min = 19 years; Max = 61 years).

The two measures used were the Work-Related Quality of Life Scale, WRQoL, developed by Van Laar et al. (2007) and validated in French by Zenasni (2011), and the QUALTRA-Scale (Salès-Wuillemin et al., 2016, 2017).

The WRQoL scale (see Supplementary Annex 1) is divided into six dimensions that structure the 24 items as follows: Job Content Satisfaction, JCS (6 items); Stress At Work, SAW (2 items), reversed for the calculations; Work Conditions Satisfaction, WCS (3 items); Control At Work, CAW (3 items); Home Work Interface, HWI (3 items); and General Well Being, GWB (6 items). The last item is not included in a dimension but measures the Overall Quality of Working Life, OQWL. Each item is coupled to a Likert-type scale going from “totally disagree” (1) to “totally agree” (5).

The QUALTRA-Scale (see Supplementary Annex 2) is composed of 52 items shared between 2 dimensions and 6 sub-dimensions. The 2 dimensions are the QWL-Perceived (26 items) and the QWL-Ideal (26 items). The 6 sub-dimensions are: Tasks and Work Organization, TaWO (3 items); Social Relationships at Work, SRW (6 items); Professional Life-Personal Life balance, PLPL (5 items); Work Environment, WE (5 items); Professional Development, PD (4 items); Recognition and Organizational Support, ReOS (5 items). Each item thus has a double assessment. To measure the QWL-Perceived, the item is coupled to a 5-point Likert-type scale, going from “never” (1) to “always” (5). To measure the QWL-Ideal, each item is assessed in terms of its usefulness in reaching an ideal QWL on a scale going from (1) “totally useless” to (5) “totally useful.” The order in which the 6 sub-dimensions appear is randomized but the measurement of the QWL-Perceived and the QWL-Ideal is always carried out in the same order. Using these two measures, the difference between the QWL-Ideal and the QWL-Perceived, called the index δ, can be calculated.

The labels of the two scales are not the same (i.e., “never” to “always” and “totally useless” to “totally useful”). It is possible, therefore, for a participant to declare being satisfied concerning a sub-dimension (e.g., Tasks and Work Organization) while at the same time considering that this dimension is not very useful. Taking this information into account makes it possible to provide more precise elements concerning the perceived quality of working life.

## 3. Results

Table 1 shows that the participants are relatively young (just over 30 years old). There are more women (*n* = 738) than men (*n* = 307) and more employees (*n* (213 + 594) = 807) than managers [*n* (92 + 137) = 229]. The majority of them come from the public sector [*n* (210 + 394) = 604].

**Table 1:** Participants demographic characteristics (*n* = 1,321).

Characteristics	Men	Women	Not specified
*n*	307	738	276
Age, M (SD)	34.3 (S.D = 12.02)	33.7 (S.D = 11.9)	34 (S.D = 11.8)
Gender % (n)	23.2%	55.8%	21%
Co-workers	213 (16.1%)	594 (44.9%)	285 (21.6%)
Managers	92 (6.9%)	137 (10.4%)	
Private sector	72 (5.4%)	304 (23%)	276 (20.9%)
Public sector	210 (15.9%)	394 (29.8%)	
Mixed sector	25 (1.9%)	40 (3%)	

### 3.1. Internal consistency of the two scales

The internal consistency of the WRQoL and the QUALTRA-Scale was first tested.

The statistical analysis (Table 2) reveals a good overall consistency of the WRQoL scale (*α* = 0.92) as the index is much higher than the cut-off value (*α* > 0.70). Moreover, no dimension has a Cronbach’s alpha lower than this value.

**Table 2:** Internal consistency of the WRQoL scale (24 items, 6 sub-dimensions).

Dimensions	Cronbach’s alpha *α*
JCS – Job Content Satisfaction (6 items)	0.82
SAW ^(1)^ – Stress at Work (2 items)	0.82
WCS – Work Conditions Satisfaction (3 items)	0.79
CAW – Control At Work (3 items)	0.77
HWI-Home Work Interface (3 items)	0.77
GWB – General Well Being (3 items)	0.88
WRQoL – 24 items	0.92

^(1)^ The answers to the items of this dimension were reversed at the time of scoring.

The statistical analysis (Table 3) reveals a good internal consistency of the QUALTRA-Scale (*α* = 0.89). Breaking it down into dimensions, it can be seen that the internal consistency of the QWL-Ideal is good (*α* = 0.88). However, there are differences between the sub-dimensions. Work Environment (*α* = 0.65) and Professional Life – Personal Life balance (*α* = 0.61) have Cronbach’s alphas below the cut-off value. For the dimension QWL-Perceived, the internal consistency is good (*α* = 0.84) and no sub-dimension has *α* below the cut-off value.

**Table 3:** Internal consistency of the QUALTRA-scale (52 items, 2 dimensions, 6 sub-dimensions).

Dimensions	Sub-dimensions	Cronbach’s alpha
QWL-Ideal	WEi – Work Environment (5 items)	0.65
	TaWOi – Tasks and Work Organization (3 items)	0.77
	PLPLi – Professional Life/Personal Life (3 items)	0.61
	SRWi – Social Relationships at Work (6 items)	0.80
	ReOSi – Recognition and Organizational Support (5 items)	0.81
	PDi – Professional Development (4 items)	0.84
	QWLi Overall Ideal (26 items)	0.88
QWL-Perceived	WEp – Work Environment (5 items)	0.76
	TaWOp – Tasks and Work Organization (3 items)	0.80
	PLPLp – Professional Life/Personal Life (3 items)	0.70
	SRWp – Social Relationships at Work (6 items)	0.88
	ReOSp – Recognition and Organizational Support (5 items)	0.82
	PDp – Professional Development (4 items)	0.85
	QWLp Overall Perceived (26 items)	0.84
QUALTRA-scale	52 Items	0.89

### 3.2. Impact of the gender and status of participants on the QWL-perceived (WRQoL and QUALTRA-Scale)

An ANOVA 2 (Gender) x 2 (Status) reveals a simple effect of the Status of respondents (Managers vs. Co-workers) on the QWL-Perceived measured by the WRQoL, *F*(6,1,203) = 5.30*, p* < 0.001*, η^2^* = 0.03; and the QUALTRA-Scale *F*(6,1,203) = 6.13, *p* < 0.001, *η^2^* = 0.04. This first result agrees with our first hypothesis. There is no effect of Gender, regardless of the scale used, which contradicts our second hypothesis. Moreover, there are no interaction effects between Gender and Status.

The dimension by dimension analysis (Table 4) within the WRQoL reveals a significant impact of Status on 5 dimensions: Job Content Satisfaction, Stress at Work, Work Conditions, and Well Being, as well as on the item measuring Overall Satisfaction.

**Table 4:** Impact of manager vs. co-worker status on the 6 dimensions of the WRQoL.

Dimensions	Managers^(1)^ *N* = 287	Co-workers^(1)^ *N* = 922	*F*(1,1,207)	*p<*
JCS – Job Content Satisfaction (6 items)	3.57 (0.05)	3.32 (0.03)	15.30	0.001***
SAW^(2)^ – Stress At Work (2 items)	2.73 (0.07)	2.82 (0.04)	13.68	0.001***
WCS – Work Conditions Satisfaction (3 items)	3.77 (0.06)	3.51 (0.03)	7.54	0.01**
CAW – Control At Work (3 items)	3.59 (0.06)	3.32 (0.03)	19.54	0.001***
HWI – Home Work Interface (3 items)	3.54 (0.06)	3.22 (0.03)	1.157	0.28
GWB – General Well Being (3 items)	3.59 (0.05)	3.41 (0.03)	14.2	0.001***
WRQoL-24 items	3.60 (0.05)	3.39 (0.03)	6.43	0.01**

^(1)^ Mean (Standard Error).

^(2)^ The answers to the items of this dimension were reversed at the time of scoring.

***p < 0.001, **p < 0.01, *p < 0.05.

Apart from the Stress At Work dimension (reversed items), the Managers have a better perceived quality of working life than the Co-workers.

The dimension by dimension analysis within the QUALTRA-Scale (Table 5) reveals a significant impact of Status. The Managers have a better perception than the Co-workers of both the Work Environment and Recognition and Organizational Support.

**Table 5:** Impact of manager vs. co-worker status on the 6 sub-dimensions of the QUALTRA-scale.

Sub-dimensions	Managers^(1)^ *N* = 287	Co-workers^(1)^ *N* = 922	*F*(1,1207)	*p<*
WEp – Work Environment (5 items)	3.79 (0.02)	3.52 (0.04)	23.54	0.001***
TaWOp – Tasks and Work Organization (3 items)	3.16 (0.05)	3.12 (0.03)	0.57	0.45
PLPLp – Professional Life/Personal Life (3 items)	3.56 (0.05)	3.57 (0.02)	0.02	0.89
SRWp – Social Relationships at Work (6 items)	3.82 (0.04)	3.83 (0.02)	0.05	0.82
ReOSp – Recognition and Organizational Support (5 items)	3.42 (0.05)	3.25 (0.03)	8.63	0.001***
PDp – Professional Development (4 items)	2.6 (0.06)	2.51 (0.03)	1.63	0.20

^(1)^ Mean (Standard Error).

***p < 0.001, **p < 0.01, *p < 0.05.

### 3.3. Impact of the gender and status of participants on the index *δ* (difference between the QWL-ideal and the QWL-perceived) of the QUALTRA-Scale

An ANOVA 2 (Gender) x 2 (Status) was carried out on the index *δ* corresponding to the difference between the QWL-Ideal and the QWL-Perceived for each item of the QUALTRA-Scale.

The analysis reveals an effect of Gender *F*(6,1,039) = 2.68, *p* < 0.02, *η^2^* = 0.01 and of Status (Managers vs. Co-workers) *F*(6,1,203) = 4.48, *p* < 0.001, *η^2^* = 0.02. However, there are no interaction effects.

The dimension by dimension analysis (Table 6) within the QUALTRA-Scale reveals a significant impact of Gender on 4 dimensions: Tasks and Work Organization, Social Relationships at Work, Recognition and Organizational Support, and Professional Development. The index *δ* is significantly higher for the Women than for the Men respondents.

**Table 6:** Impact of gender on the index *δ* for the 6 dimensions of the QUALTRA-scale.

Sub-dimensions	Women^(1)^ *N* = 738	Men^(1)^ *N* = 307	*F*(1,1043)	*p<*
δ WEp – Work Environment (5 items)	0.74 (0.03)	0.67 (0.01)	1.36	0.24
δ TaWOp – Tasks and Work Organization (3 items)	1.46 (0.04)	1.29 (0.06)	4.70	0.03*
δ PLPLp – Professional Life/Personal Life (3 items)	0.86 (0.03)	0.79 (0.01)	0.96	0.33
δ SRWp – Social Relationships at Work (6 items)	0.75 (0.03)	0.61 (0.04)	7.13	0.008**
δ ReOSp – Recognition and Organizational Support (5 items)	1.28 (0.03)	1.09 (0.01)	7.63	0.006**
δ PDp – Professional Development (4 items)	1.85 (0.03)	1.60 (0.01)	10.53	0.001***

^(1)^ Mean (Standard Error).

***p < 0.001, **p < 0.01, *p < 0.05.

The sub-dimension by sub-dimension analysis (Table 7) within the QUALTRA-Scale reveals a significant impact of Status on: Work Environment, Tasks and Work Organization, Recognition and Organizational Support and Professional Development. The difference between the ideal and the perceived is significantly higher for the Co-workers than for the Managers.

**Table 7:** Impact of manager vs. co-worker status on the index *δ* for the 6 dimensions of the QUALTRA-scale.

Dimensions	Managers^(1)^ *N* = 287	Co-workers^(1)^ *N* = 922	*F*(1,1208)	*p<*
*δ* WEp – Work Environment (5 items)	0.49 (0.05)	0.80 (0.03)	23.90	0.001***
*δ* TaWOp – Tasks and Work Organization (3 items)	1.29 (0.06)	1.44 (0.03)	4.33	0.04*
*δ* PLPLp – Professional Life/Personal Life (3 items)	0.76 (0.05)	0.86 (0.03)	2.92	0.08
*δ* SRWp – Social Relationships at Work (6 items)	0.68 (0.04)	0.73 (0.02)	0.80	0.37
*δ* ReOSp – Recognition and Organizational Support (5 items)	1.08 (0.05)	1.29 (0.03)	9.99	0.002**
*δ* PDp – Professional Development (4 items)	1.67 (0.06)	1.82 (0.03)	3.86	0.05*

^(1)^ Mean (Standard Error).

***p < 0.001, **p < 0.01, *p < 0.05.

In order to complete this analysis, the impact of the public/private sector was examined. In effect, given the different rules regarding career development, salaries and working conditions, this characteristic may have had an impact on the answers given.

An ANOVA 2 (Gender) x 2 (Status) x 2 (Sector) was carried out on a subpopulation (*n* = 980) within our sample. This analysis confirms the simple effect of the Status of respondents (Managers vs. Co-workers) *F*(18,940) = 1.96, *p* < 0.01, *η^2^* = 0.001. There are no effects of Gender or Sector and no interaction effects.

## 4. Discussion

This study shows an impact of status on the perceived QWL. For most of the dimensions of the QWL, managers report a better QWL than the co-workers, particularly for the professional life/personal life balance, control at work, working conditions, the work environment and the feeling of recognition and organizational support. These results agree with our second hypothesis. In fact, although managers experience stress at work, they also have a better perceived quality of working life than the co-workers. Managers occupy a special position in the work organization. This can be reflected in the possibility of having a personalized space (an office, for example). Their tasks also lead them to work with a high degree of autonomy and to be recognized by their teams as well as by their hierarchy. These three elements confirm the studies conducted on the subject.

In contrast, this study does not show an effect of gender on the perceived QWL, contradicting our first hypothesis. In this study, the women do not report a worse QWL than the men, regardless of the scale used. This result could be questioned, especially the absence of difference between men and women for the professional life/personal life dimension, even though this has often been confirmed in the literature.

Considering the index *δ* that measures the difference between the QWL-Ideal and the QWL-Perceived, there is an effect of both gender and status, with a great similarity in the response profiles between the women and the co-workers. In these two groups, compared to the men and the managers respectively, the index *δ* is higher for 3 of the 6 sub-dimensions of the QWL: Tasks and Work Organization, Recognition and Organizational Support and Professional Development. In addition, more specifically, the index *δ* in the answers of women is higher for the sub-dimension Social Relationships at Work whereas for the co-workers, it is higher for the sub-dimension Work Environment.

It emerges from this analysis that if the index *δ* is higher, it is because of the higher expectations among women and among co-workers concerning these 3 sub-dimensions of QWL and this regardless of the sector of activity (public or private).

Women and employees therefore consider important (1) relationships at work, i.e., the possibility of talking to colleagues, having friendly relationships with them and being part of a team, as well as being able to rely on them in case of difficulty; (2) recognition and organizational support from the hierarchy, i.e., having managerial confidence and not being pressured. Finally, (3) they also expect opportunities for job advancement, i.e., being able to move up in new assignments and projects or in hierarchical levels.

Managers who can create a positive work environment, by introducing, for example, women-friendly policies, should be able to improve women’s perceived quality of working life and, more generally, contribute to reducing gender inequality at work.

Although our sample size was sufficient, a large number of factors could have affected the results and it is difficult to control them all (Casper et al., 2007). For example, in our study, the women constitute 55.8% of our sample compared to men 23.2% (21% of the sample did not reply to the question on gender). The women co-workers constituted 44.9% of our sample compared to 16.1% of men whereas the women managers were 10.4% of our sample compared to 6.9% of men (21.6% of the sample did not reply to the question on status). These differences could have had an impact on the analysis.

Our results are however interesting; they make an innovative contribution to the measurement of the QWL. These double measurements have two advantages. From an academic perspective, they make it possible to understand the way in which individuals construct their subjective perceptions. These results differentiate two dimensions in the evaluation that individuals make of their working conditions. First, there is what they perceive and evaluate, and second, what they hope for and what they tend toward. It is this movement back and forth between the two that allows people to assess their working conditions. In accordance with Gilgeous (1998), it is then possible to measure an individual’s satisfaction with his/her current working life dimensions in comparison with his/her pursued or ideal quality of working life.

Concerning the practical implications of this study, our results underline the importance of including a measure of the QWL expected (ideal) and not just the perceived QWL. Thanks to this dual measurement, it is possible not only to consider satisfaction with the current working conditions but also to understand the working conditions that are expected by employees. Thus, the diagnosis of the QWL can be accompanied by an action plan based on the prioritized dimensions.

The study is not yet finished. The measure should be extended to a larger sample of female managers. In addition, the impact of other factors should be explored, such as the situation in relation to employment (for example, fixed or fixed-term contract; part-time or full-time contract). This work is scheduled for the next 6 months.

## 5. Conclusion

These results suggest the hypothesis of a greater frustration among women and co-workers due to their expectations regarding different dimensions of the quality of working life. It would therefore be beneficial to provide support to work teams and management in relation to these issues.

Although our study is not based on a representative sample, it reveals two notable profiles, women and co-workers. Women are particularly affected by social relationships at work, an issue that has not yet been explored to our knowledge. This deserves further investigation, as does the professional development of co-workers,

A number of courses of action could be implemented in the workplace, which must be supported by an internal or external occupational psychologist. Clearly, they will require a great deal of collaboration and cooperation (Mirvis and Lawler, 1984).

In concrete terms, this work must be carried out not only with the employees but also in relation to the social partners, the occupational health service, and the training and human resources departments, to implement an approach to the quality of working life that is adapted to expectations.

With regard to helping organizations improve social relationships at work, this could be achieved by training (managers and co-workers) and/or by awareness days on such topics as non-violent communication or the use of emails, including real cases for discussion. It could also take place during interdepartmental meetings, in the form of focus groups of just managers or just co-workers, and based on feedback from within and outside the department.

Concerning professional development, this could be improved by monitoring careers within the organization. For example, it would be useful to go beyond the feedback provided by the annual interview, which measures the expectations of employees in terms of training. Regular professional progress reviews could be put in place, including a skills review planned at agreed intervals. This monitoring would provide an update on the professional development expectations of employees and would also constitute a valuable support for the HR department.

These courses of action and many others have a vital role to play in the QWL approach and should be adapted according to the nature of the workplace.

## Data availability statement

The raw data supporting the conclusions of this article will be made available by the authors, without undue reservation.

## Ethics statement

Ethical review and approval was not required for the study on human participants in accordance with the local legislation and institutional requirements. The patients/participants provided their written informed consent to participate in this study.

## Author contributions

ES-W: questionnaire conception, statistical analysis, and article writing. BM-K, JC, MG, and AD: questionnaire diffusion and articleproof read. MG and AD: questionnaire distribution and article proofread. All authors contributed to the article and approved the submitted version.

## Conflict of interest

The authors declare that the research was conducted in the absence of any commercial or financial relationships that could be construed as a potential conflict of interest.

## Publisher’s note

All claims expressed in this article are solely those of the authors and do not necessarily represent those of their affiliated organizations, or those of the publisher, the editors and the reviewers. Any product that may be evaluated in this article, or claim that may be made by its manufacturer, is not guaranteed or endorsed by the publisher.
