# Supplementary material for: The quality of working life: gap between perception and idealization impact of gender and status
Source: Front Psychol. 2023 May 19;14:1112737. doi: 10.3389/fpsyg.2023.1112737 (PMC10235489; doi:10.3389/fpsyg.2023.1112737)
Supplement: Supplementary file 1 [file Data_Sheet_1.docx]

**Annex 1.**

WRQoL Scale

Instructions given to participants: This questionnaire is designed to assess your quality of working life. Please do not take too long over each question; we want your first reaction not a long drawn out thought process. Please do not omit any questions. This isn’t a test, simply a measure of your attitudes to the factors that influence your experience at work.

*Please indicate your answers by putting a cross in the appropriate case*

|  | Strongly Disagree | Disagree | Neutral | Agree | Strongly Agree |
| --- | --- | --- | --- | --- | --- |
| **1.** I have a clear set of goals and aims to enable me to do my job [JCS] |  |  |  |  |  |
| **2.** I feel able to voice opinions and influence changes in my area of work [CAW] |  |  |  |  |  |
| **3.** I have the opportunity to use my abilities at work [JCS] |  |  |  |  |  |
| **4.** I feel well at the moment [GWB] |  |  |  |  |  |
| **5** My employer provides adequate facilities and flexibility for me to fit work in around my family life [HWI] |  |  |  |  |  |
| **6.** My current working hours / patterns suit my personal circumstances [HWI] |  |  |  |  |  |
| **7.** I often feel under pressure at work [INVERSE] [SAW] |  |  |  |  |  |
| **8.** When I have done a good job it is acknowledged by my line manager [JCS] |  |  |  |  |  |
| **9.** Recently, I have been feeling unhappy and depressed [INVERSE] [GWB] |  |  |  |  |  |
| **10.** I am satisfied with my life [GWB] |  |  |  |  |  |
| **11.** I am encouraged to develop new skills [JCS] |  |  |  |  |  |
| **12.** I am involved in decisions that affect me in my own area of work [CAW] |  |  |  |  |  |
| **13.** My employer provides me with what I need to do my job effectively [WCS] |  |  |  |  |  |
| **14.** My line manager actively promotes flexible working hours / patterns [HWI] |  |  |  |  |  |
| **15.** In most ways my life is close to ideal [GWB] |  |  |  |  |  |
| **16.** I work in a safe environment [WCS] |  |  |  |  |  |
| **17.** Generally things work out well for me [GWB] |  |  |  |  |  |
| **18.** I am satisfied with the career opportunities available for me here [JCS] |  |  |  |  |  |
| **19.** I often feel excessive levels of stress at work [INVERSE] [SAW] |  |  |  |  |  |
| **20.** I am satisfied with the training I receive in order to perform my present job [JCS] |  |  |  |  |  |
| **21.** Recently, I have been feeling reasonably happy all things considered [GWB] |  |  |  |  |  |
| **22.** The working conditions are satisfactory [WCS] |  |  |  |  |  |
| **23.** I am involved in decisions that affect members of the public in my own area of work [CAW] |  |  |  |  |  |
| **24.** I am satisfied with the overall quality of my working life |  |  |  |  |  |

*Footnote*: 6 dimensions of QW are quoted, *[CAW]* Control at Work; *[GWB]* General Well Being, *[HWI]* Home Work Interface; *[JCS]* Job Content Satisfaction; *[SAW]* Stress at Work; *[WCS]* Working conditions; *[OVL]* overall QWL

**Annex 2.**

QUALTRA-Scale (QS-27)

Instructions given to participants: The questions below concern your perception of your Quality of Life at Work. They are grouped into several themes (your work environment, your tasks and the organization of your work, the link between your professional and private life, your social relations at work, your feeling of recognition and your professional development).

For each theme there are two versions. In the first version (version A), you must say whether the proposals made can be found in your *current* work; then in the second version (version B) what is for you the interest, in other words the *importance* of these proposals *for a good quality of life at work*.

*Please, put a cross in the appropriate box, from 1 to 5*

| 1^A^- About your current work environment you consider that … | Never (1) | Rarely (2) | Sometimes (3) | Often (4) | All the time (5) |
| --- | --- | --- | --- | --- | --- |
| 1. You have equipment that works properly |  |  |  |  |  |
| 2. You have equipment adapted to your work |  |  |  |  |  |
| 3. You have a satisfying room temperature (Not too high not too low) |  |  |  |  |  |
| 4. You have a sufficiently spacious rest room |  |  |  |  |  |

| 1^B^ – Concerning work environment What is your position concerning the interest of the following proposals for having a good quality of life at work … | Totally useless (1) | Somewhat useless (2) | Neither useful nor useless (3) | Somewhat useful (4) | Totally useful (5) |
| --- | --- | --- | --- | --- | --- |
| 1. To have equipment that works properly |  |  |  |  |  |
| 2. To have equipment adapted to work |  |  |  |  |  |
| 3. To have a satisfying room temperature (Not too high not too low) |  |  |  |  |  |
| 4. To have a sufficiently spacious rest room |  |  |  |  |  |

| 2^A^- About your current tasks and work organization you consider that … | Never (1) | Rarely (2) | Sometimes (3) | Often (4) | All the time (5) |
| --- | --- | --- | --- | --- | --- |
| 1. You have enough time to complete your tasks or missions |  |  |  |  |  |
| 2. You have the possibility to manage your time without being interrupted |  |  |  |  |  |
| 3. You have very specific tasks or missions, everyone is in his place and knows what he has to do |  |  |  |  |  |
| 4. You have a reasonable amount of work |  |  |  |  |  |

| 2^B^ – Concerning tasks and work organization, what is your position concerning the interest of the following proposals for having a good quality of life at work … | Totally useless (1) | Somewhat useless (2) | Neither useful nor useless (3) | Somewhat useful (4) | Totally useful (5) |
| --- | --- | --- | --- | --- | --- |
| 1. To have enough time to complete tasks or missions |  |  |  |  |  |
| 2. To have the possibility to manage time without being interrupted |  |  |  |  |  |
| 3. To have very specific tasks or missions, everyone is in his place and knows what he has to do |  |  |  |  |  |
| 4. To have a reasonable amount of work |  |  |  |  |  |

| 3^A^- About the link between your professional and personal life in your current job you consider that … | Never (1) | Rarely (2) | Sometimes (3) | Often (4) | All the time (5) |
| --- | --- | --- | --- | --- | --- |
| 1. You are able to put your personal life before your professional life |  |  |  |  |  |
| 2. You are able to separate your private life from your professional life |  |  |  |  |  |
| 3. You have a rhythm of life/work compatible with your family life |  |  |  |  |  |

| 3^B^ – Concerning the link between professional and personal life what is your position concerning the interest of the following proposals for having a good quality of life at work … | Totally useless (1) | Somewhat useless (2) | Neither useful nor useless (3) | Somewhat useful (4) | Totally useful (5) |
| --- | --- | --- | --- | --- | --- |
| 1. To put personal life before your professional life |  |  |  |  |  |
| 2. To separate private life from professional life |  |  |  |  |  |
| 3. To have a rhythm of life/work compatible with family life |  |  |  |  |  |

| 4^A^- About the social relations in your current job, you consider that…. | Never (1) | Rarely (2) | Sometimes (3) | Often (4) | All the time (5) |
| --- | --- | --- | --- | --- | --- |
| 1. You have friendly relations with your colleagues |  |  |  |  |  |
| 2. You have the feeling of being part of a team |  |  |  |  |  |
| 3. You have the possibility to dialogue with your colleagues |  |  |  |  |  |
| 4. You have good communication with your colleagues |  |  |  |  |  |
| 5. You have a good work atmosphere |  |  |  |  |  |
| 6. You have the support of your colleagues in difficult situations |  |  |  |  |  |

| 4^B^ – Concerning social relations what is your position concerning the interest of the following proposals for having a good quality of life at work … | Totally useless (1) | Somewhat useless (2) | Neither useful nor useless (3) | Somewhat useful (4) | Totally useful (5) |
| --- | --- | --- | --- | --- | --- |
| 1. To have friendly relations with colleagues |  |  |  |  |  |
| 2. To have the feeling of being part of a team |  |  |  |  |  |
| 3. To have the possibility to dialogue with colleagues |  |  |  |  |  |
| 4. To have good communication with colleagues |  |  |  |  |  |
| 5. To have a good work atmosphere |  |  |  |  |  |
| 6. To have the support of colleagues in difficult situations |  |  |  |  |  |

| 5^A^- About your feeling of recognition in your current job, you consider | Never (1) | Rarely (2) | Sometimes (3) | Often (4) | All the time (5) |
| --- | --- | --- | --- | --- | --- |
| 1. You have a financial recognition or bonus |  |  |  |  |  |
| 2. You have the confidence of your hierarchical superiors who give you responsibilities |  |  |  |  |  |
| 3. You have the help of your hierarchical superiors in case of need |  |  |  |  |  |
| 4. You have the support of your immediate superior (n+1) who defends you in case of conflict |  |  |  |  |  |
| 5. You are not pressured by your hierarchy |  |  |  |  |  |

| 5^B^ – Concerning the feeling of recognition, what is your position concerning the interest of the following proposals for having a good quality of life at work … | Totally useless (1) | Somewhat useless (2) | Neither useful nor useless (3) | Somewhat useful (4) | Totally useful (5) |
| --- | --- | --- | --- | --- | --- |
| 1. To have a financial recognition or bonus |  |  |  |  |  |
| 2. To have the confidence of the hierarchical superiors who give responsibilities |  |  |  |  |  |
| 3. To have the help of the hierarchical superiors in case of need |  |  |  |  |  |
| 4. To have the support of the immediate superior (n+1) who defends in case of conflict |  |  |  |  |  |
| 5. Not to be pressured by the hierarchy |  |  |  |  |  |

| 6^A^- About your possibilities of evolution in your current job, you consider … | Never (1) | Rarely (2) | Sometimes (3) | Often (4) | All the time (5) |
| --- | --- | --- | --- | --- | --- |
| 1. You can change of position for moving up in the hierarchy |  |  |  |  |  |
| 2. You can leave your position to join another one of the same hierarchical level but with other skills and responsibilities |  |  |  |  |  |
| 3. You can change position without necessarily changing location |  |  |  |  |  |
| 4. You can easily change of job in order to develop your skills |  |  |  |  |  |
| 5. You can increase your salary while still being in the same position and at the same hierarchical level |  |  |  |  |  |

| 6^B^ – Concerning possibilities of evolution what is your position concerning the interest of the following proposals for having a good quality of life at work … | Totally useless (1) | Somewhat useless (2) | Neither useful nor useless (3) | Somewhat useful (4) | Totally useful (5) |
| --- | --- | --- | --- | --- | --- |
| 1. To change of position for moving up in the hierarchy |  |  |  |  |  |
| 2. To leave a position to join another one of the same hierarchical level but with other skills and responsibilities |  |  |  |  |  |
| 3. To change position without necessarily changing location |  |  |  |  |  |
| 4. To easily change of job in order to develop your skills |  |  |  |  |  |
| 5. To increase salary while still being in the same position and at the same hierarchical level |  |  |  |  |  |

In summary, you consider your current Quality of Life at Work to be... (Circle the appropriate number)

| Not at all satisfactory |  |  |  | Totally satisfactory |
| --- | --- | --- | --- | --- |
| 1 | 2 | 3 | 4 | 5 |
